# New York Academy of Medicine

**Published:** 1855-11

**Authors:** 


New York Academy of Medicine.—On motion of the Secretary, Dr. Stone, of New Orleans, being present, was requested to give the Academy his views respecting yellow fever.

Dr. Stone, after an expression of thanks for the honor conferred upon him, proceeded to say, that in his opinion, yellow fever is a specific disease, the same in all latitudes and all longitudes, unmodified by topographical causes or changes of climate, but under all circumstances the same, identical and unchanged. He is not prepared to say that one cannot have it a second time, but these cases are very rare; for during a twenty-three years' residence in New Orleans, he has not seen a single instance. Those who believe in second attacks call other diseases yellow fever, as the bilious fever of the South, etc. When that fever is epidemic anything which disturbs the system develops it; at such seasons it is impossible to have any other disease. At such times many have the disease in a light manner—known to be such by the symptoms peculiar to its convalescence—yet such never have it again. Even accidents and injuries occurring at such times are sure to be followed by yellow fever, in from twenty-four to thirty-six hours.

The young Creoles are, many of them, educated in Paris, but after many years, ten or more, of absence, on their return do not have it, if they have been acclimated before their departure. All children have to be acclimated, but the disease may run out in several generations—it certainly is less virulent.

He has often noticed the fever in children in a house where the yellow fever was; but fortunately the disease was not suspected in them, and so they were let alone, and rarely required medicine.

Many attempts have been made by statistics to discover its *cause*, but, like cholera, it escapes observation. A warm climate is an essential element. A continued heat at a certain high degree was once supposed to be essential, but this is now disbelieved, for in 1847 it commenced early; in 1853 earlier, say in latter part of May, and June, when there was no steady heat; and we had a remarkably cold spring that year. This year he saw the pure and genuine fever on the 30th April. It occurred in persons coming from Pittsfield, and residing in unhealthy locations of the city. Moisture seemed not essential, for it raged equally in the high lands as the low—where the dry trade-winds blow, or where the air was damp. It is true that when it appears the rain usually commences; but New Orleans has daily showers at certain seasons, and yet without any disease. Dr. G. has seen three seasons in succession when there were all the peculiarities of the weather that are said to produce the fever—heat, moisture, etc.—present, when the season proved remarkably healthy. This year it was very dry, and the sugar cane died from want of moisture, and all were suffocated by dust, when the disease first appeared.

Many who investigate yellow fever, form theories, and afterwards hunt for facts, and they are apt to get hold of instances which favor their theories; and these, when arranged in a catalogue, appear quite formidable; but when investigated quite a different result is obtained. The very fact of its being identical in all latitudes, and varying so in regard to moisture, is sufficient proof that they have got at the causes, and that it is something which will probably never be remedied by art. There is no doubt that a filthy and unhealthy city will serve to spread the disease; whatever weakens the vital powers favors its spread. It does not, however, appear to give virulence to the disease—indeed, it appears in climates that are almost perfectly healthy, where there are no remittents or intermittents, nothing but accidental sickness.

As to its *mode of propagation*, some believe in its contagion; but it is incapable of generating its own poison under any circumstances. Were it so, being such a specific, marked and formidable disease, it could not but be evident. It is all around us, and we cannot so well observe as in smaller districts, where this fact is plainly to be proved. A vessel from Bremen with emigrants, came from the South side of Cuba, and when a hundred miles from land, they took the fever, and many died. On landing some thirty were sent to the hospital, and put into the wards with the unacclimated, and many died—but no one took the fever. I did not even name its presence, for I knew it would not spread, as there was no epidemic atmosphere; and more than a month occurred before this epidemic did come. This was in June. 1849, and the first case originated in the city in July, and it was not till August that it could be said to be epidemic. When the Charity Hospital was moved, the first season that Dr. S. was at New Orleans, the house was crowded, and beds were made on the floors and in the entries, etc. Many died of yellow fever, the beds covered with excrements laid in the passage, indeed the filth was so great as to generate house-fever, but there was no epidemic in that neighborhood, and those lying around—the unacclimated—did not take the disease. There were many who took the house-fever, but escaped the yellow fever, which did not even mark the other diseases in the house with its own type.

There, again, the hospital was out upon a swamp, but the inhabitants around were comparatively more exempt than those who labored in various parts of the city.

The steamboats are said to carry the disease from the city to the various stopping places up the river, but when they get above Vicksburg it never takes. Fifty cases were left at Memphis by a boat in 1847, and yet it has never been known there before this year. In most of the cases you will find it at these places before the steamboat lands. They were landed at Memphis in the second and third stages of the disease. It was not till 1853 that it was supposed to be contagious, or, rather, infectious, terms which Dr. S. considers synonymous. Dr. S. also stated other instances in proof of its non-contagious character, and remarked that he believed that if all the facts which seem to prove the contagious properties of the disease were properly investigated, we should come to the conclusion that contagion has no effect whatever in increasing the number of victims to this disease.

Dr. S. thinks that *moral causes* have some influence in producing the disease; as, for instance, the moral effect of one case occurring in a family is sufficient to cause all other members to take it—but only in the yellow-fever region. Any excitement at such times is sufficient to create or develop it. It was noticeable that among the unacclimated—the Northerners and others who united together for self-protection—the nurses and assistants were the last to take it; while the timid, who shunned infected localities, who sneaked off to bed, who feared the night air, who deprived themselves of exercise by their seclusion, were, by these mental causes, the first to take it. In Norfolk, recently, it was believed at first, not to be personally contagious, but all felt that they were shut up, obliged to stay and perish, and the moral effect was disadvantageous. It was difficult to get nurses to attend upon the sick. They did, however, in some instances, attend to their own relatives, but others were unnursed and neglected. Dr. S. thinks that the alarm which existed increased very much the number of fatal cases.

As to the *pathology* of yellow fever, Dr. Stone says that it has literally no anatomical character—it is a blood poison. In yellow fever proper there are no traces left to account for symptoms or death. Occasionally there are engorgements from the sequelæ—but none to account for the black vomit, etc. We must give up the idea of mucous inflammations. Dr. S. could never find anything after death that could account for a single symptom that could cause death. The fact of the mucous membrane being soft is accidental—it depends entirely upon the time and manner of death, and secretions of the stomach. Black vomit possesses no marked character to distinguish it from ordinary blood. Dr. S. once made some in the apothecary's shop, which was believed by the best observers to be black vomit proper. He thinks that the vomiting in this disease is merely nervous. There is, in fact, no irritability or tenderness of the stomach, but simply heightened nervous sensibility.

The straw color of the liver described by Louis, is owing to nothing more than a change in the fluids generally. The change in the hue of the skin is a little more manifest in the liver, which, in a healthy condition, is nearer the color of the blood. It is not the color of jaundice, but is of a dirty yellow, which is well represented around an ecchymosis when a change is taking place in the fluids through the influence of absorption.

*Treatment.*—Yellow fever is a self-limited disease; it is not to be treated—it is to be managed. All that is to be done is to keep the patient alive for a certain time, and he will get well.

The disease is ushered in with a chill or slight rigor, often scarcely noticeable, followed by heat in the forehead, pain in head, limbs and back. This is again followed by a hot fever, and if the patient be kept under cover, and carefully treated, these symptoms will quietly terminate in two or three days; but if left to themselves to toss about and not remain under cover, the sweating stage passes on for five or six days, and collapse, black vomit and death result.

The only treatment found by Dr. S. to be useful, is to favor the efforts of nature in prolonged sweating, calm and rest of the system, and the fever will generally be got rid of in the first two or three days. It terminates in favorable cases on or before the third day, leaving the skin natural. Those who treat it otherwise than expectantly do not understand the nature of the disease.

Among those who may be said to understand the disease, there are two methods of treatment: the expectant—cups to temples to relieve cephalalgia, slight laxatives to open the bowels, hot baths under the bed-clothes. Others give quinine. Dr. S. was the first to do so, whoever has the credit, but no matter about that. The only difference is that they do not give it with any specific object. His method was a full dose at the beginning of the disease, but not afterward. Thus given it promoted and prolonged the sweating stage, and while this was kept up the patient was safe. It was remarked that they would get well without quinine where it was generally prescribed. He was physician for many years to a hospital where there were forty to fifty cases a day, and he noted that those in favor of this quinine treatment were about 10 per ct.

Dr. S. remarked that in 1847 he treated forty cases in succession by quinine, among mechanics, who had no nursing except what was provided by friends, and did not lose a single patient. It was in a favorable epidemic, but considering their destitution of proper nursing, deaths would have been as likely to occur as in a worse epidemic.

As to the use of *calomel* in this disease, there is no possible condition of the system where there could be any benefit derived from its use—there was no local disease. The liver has nothing to do with it. He knew this, for he had followed the patients of the Calomelites to the dead house in plenty. While serving as surgeon of the Charity Hospital, the medical side of the hospital was full, and there were several mechanics who applied for admittance, and wished to be treated by Dr. S.; and as he had some empty beds, he received them. But one was sent to the medical wards, and he gave him foot-baths, kept him warm, regulated his drinks, etc., and gave quinine. The attending physician came in the morning; he ordered a drachm of calomel, but Dr. S. put up magnesia. The next day he was better, and the doctor repeated the dose, but he took the liberty to repeat the magnesia. He got well, and thenceforward was one of the attending physician's 'brag cases,' for he dared not tell him of his doings. Then came new physicians from Paris, full of Broussais' theory, and they bled and boasted of their successes also for a while, and in truth they did succeed as well as the former. Then came Eclectics with fanciful theories; they gave a little calomel to disgorge the liver a little, cups a little, leeches a little, end with a result very little different from the others. In fact some will get well in spite of the treatment, and then again some forms of the fever are fatal in the beginning. When, however, they are brought side by side, and we can observe them *en masse*, we can more properly judge of the relative value of the different modes of treatment than in the isolated cases met with in ordinary practice. The difference is very manifest—all perturbating treatment is alike bad.

There are some peculiarities in the disease that might not at first strike one—the disturbed nervous system, and especially delirium—one of the worst symptoms. This may appear at first, but not usually. Its first evidence is restlessness and want of sleep; objects are seen very much as in *mania-a-potu*. Narcotics produce stupor and death, for the patients with this disease are peculiarly susceptible to morphine; stimulants are much better. You must watch to give the stimulants as early as possible; they then sweat off, and are relieved in twenty-four to thirty-six hours; but even then they must not be disturbed—if raised up they faint away. Perfect and absolute rest, body and mind, are indispensable. If they are excited the heat returns, and they die. Watch for sleeplessness, and give minute anodynes and stimulants, such as they are able to bear. Of those cases that run on and approach the period of black vomit, if managed properly as to drinks, by avoiding bitter infusions, etc., very many recover; and those that do not vomit would have the black vomit if they vomited at all. Some have a preference for certain kinds of drink, as porter; others prefer brandy, etc. Give those agreeable to the palate. As they approach the black-vomit period with previous restlessness and acid secretions, give some alkali, with minute doses (say a 20th or 30th of a grain) of morphine, with champagne, ale, beef-essence, etc. Impart to the patient a feeling of safety and security. And yet I thought in proportion to the mildness of the disease was the danger; for quiet is absolutely necessary, and coercion does not answer. The patient is to be managed, not treated.

Foot-baths under the clothes will often produce favorable sweats. When in a state of dry heat forced perspiration is bad; sponging with tepid water is then better. The douche is but of temporary benefit, and the subsequent reaction leaves the patient worse. Sponging with lemon-juice, sweet-oil and salt is used by the Creoles and Spaniards, but pure water is better. All that is to be done is to ease them through.

Dr. Stone concluded by remarking that he had given the prominent heads of treatment, with little amplitude, for fear of fatiguing the Academy. He proposed, however, to write it down, and publish it in some magazine.

Dr. Gardner inquired in what sense Dr. Stone used the word 'acclimation?'

Dr. Stone replied that it meant simply having had the fever. No one was secure till he had had it.

Dr. White then referred to the communication read before the Academy some years since on the same subject by Dr. A. Smith, of Texas, and published in the first volume of the Transactions of the Academy, and remarked upon the similarity of his views with those of Dr. Stone. Dr. Smith then gave a graphic description of the difference between yellow fever and bilious fever, and among the pathognomic symptoms, stated that yellow fever was characterized by only one paroxysm. Dr. Smith and Dr. Stone also agree as to the treatment, both being opposed to the perturbing practice, and both advocate careful nursing, and the 'expectant' plan. Dr. W. wished to ask whether the observations of Dr. Stone corroborate the statement of Dr. Smith that yellow fever is uniformly characterized by one paroxysm only?

Dr. Stone replied that there is literally but one paroxysm; but if convalescence be disturbed, there is a return of the first stage which may be called a relapse.

Dr. Gardner stated that Dr. Ashbel Smith laid great stress on covering, and said the patient should be enveloped in blankets and carefully excluded from the air. Was this correct, and what drinks are proper?

Dr. Stone.—Careful covering of the entire body and limbs is absolutely requisite, but not to swelter under too much covering. If the hands were but exposed, sometimes the heat would return and a relapse ensue. Some mild diaphoretics may be given; such drinks as the patients desire; one year all want brandy and water, other years malt liquors. Give that which is desired, and carefully avoid even the nervous shock caused by a bitter or disagreeable medicine. Sponging the body under the clothes, ice-water to head, generally were followed by reaction and more pain. Dr. Cartwright had pursued the opposite plan of enveloping the head in warm fomentations.

Dr. Detmold wished to ask a few questions: 1. If there was no anatomical change observal after death by yellow fever as hemorrhage, etc.? 2. Was there in the South any popular or scientific belief that the appearance of frost arrested the progress of the disease? 3. If the 'breaking bulk' on board the vessel supposed to have imported the disease into Norfolk had anything to do with the breaking out of the disease there?

Dr. Stone replied: 1st. The black vomit left no trace behind. There were some who, making examinations, always saw just what they wanted to; others were unaccustomed and were deceived, thinking they saw softening, etc., when they saw only ordinary conditions, perhaps slightly modified. The black vomit, no doubt, was an hemorrhage; but it all washed off, leaving the stomach redder or paler, as it appeared in other diseases. The yellow liver he regarded as merely an exaggeration of the same tint observed on the skin and elsewhere. 2d. Frost does not check the disease. As a general rule, when the epidemic came early it left early, and when late it left late. In 1847 it was cut short in mid-summer. The disease has never renewed after it has ceased, by the return of people from their summer retreats, as it would if contagious. 3d. As to the breaking of bulk of ship in

Norfolk, he had made many investigations, but could only obtain second-hand information. Dr. Upshur, now dead, informed him that the yellow fever was in Gosport before the arrival of the Franklin, and that this vessel had no yellow fever on board, and was twenty-eight days in quarantine.

The Norfolk epidemic was the identical yellow fever seen the same in every locality, but in a severer form than ordinary. It first commenced at Rio in 1851, then spread throughout Brazil, Para, northern part of South America, going into the country and the small villages; into the West Indies, into the plantations heretofore unknown to be ever affected, attacking negroes (generally enjoying immunity;) into the pine woods of Alabama, and the heights between this State and Georgia; the next year throughout Georgia and South Carolina; this year in Memphis, where it never was before epidemic, and Norfolk. It is creeping over the country, and there is some reason to fear (why, cannot be said) that next year it may reach New York.

Dr. Corson wished to know the doses of quinine which Dr. S. gave, and the vehicle.

Dr. Stone replied that he usually gave a single dose of fifteen or twenty grains, according to circumstances, at the outset, perhaps ten grains more twelve hours after, but none unless on the first day; and the second day it is entirely useless, and after that actually injurious, although they bear it better than any other remedy. It causes vomiting when given late, and is not necessary, for its effects last several hours after its administration.

Dr. S. regretted the necessarily imperfect and desultory manner in which he had been obliged to make his remarks, but he hoped that on drawing up his paper, some weeks hence, he might be enabled to make it more consecutive and logical. He thanked the Academy for the honor they had done him in hearing him.—*New York Medical Times.*

